# Change in self-rated general health is associated with perceived illness burden: a 1-year follow up of patients newly diagnosed with type 2 diabetes

**DOI:** 10.1186/s12889-015-1790-6

**Published:** 2015-04-30

**Authors:** Anni Brit Sternhagen Nielsen, Per Jensen, Dorte Gannik, Susanne Reventlow, Hanne Hollnagel, Niels de Fine Olivarius

**Affiliations:** The Research Unit for General Practice and Section of General Practice, Department of Public Health, University of Copenhagen, Copenhagen, Denmark; Section of Biostatistics, University of Copenhagen, Copenhagen, Denmark

**Keywords:** Adaptation, Coping, Psychological, Diabetes mellitus, type 2, Family practice, Follow-up studies, Glycaemic control, Health behaviour, Illness burden, Self-rated general health

## Abstract

**Background:**

Diabetic patients’ lifestyle adaptations to improve glycaemic control are not always followed by improvements in self-rated general health (SRH). The perceived impact of diabetes on patients’ daily lives may influence changes in their SRH. This paper examines the association of illness severity, treatment, behavioural, and coping-related factors with changes in SRH from diagnosis of type 2 diabetes until one year later, in a population-based sample of 599 patients aged 40 years or over who were treated in general practice.

**Methods:**

Change in SRH was estimated by a cumulative probit model with the inclusion of covariates related to SRH (e.g. illness severity at diagnosis, behaviour, treatment, and the perceived impact of diabetes on patients’ daily lives one year later).

**Results:**

At diagnosis, 11.6% of patients reported very good, 35.1% good, 44.6% fair and 8.5% poor SRH. Physical inactivity, many diabetes-related symptoms, and cardiovascular disease were related to lower SRH ratings. On average SRH improved by 0.46 (95% CI: 0.37; 0.55) during the first year after diagnosis without inclusion of covariates. Mental and practical illness burden was the only factor associated with change in SRH, independent of patients’ diabetes severity and medical treatment (p = 0.03, multivariate analysis). Compared to otherwise similar patients without illness burden, increase in SRH was marginally smaller among patients who expressed minor illness burden, but much smaller among patients with more pronounced illness burden.

**Conclusions:**

Much as one would expect, many patients increased their SRH during the first year after diabetes diagnosis. This increase in SRH was not associated with indicators of illness severity or factors reflecting socio-demographic circumstances, but patients experiencing illness burden had a smaller increase than those who reported no illness burden. We suggest that during the diabetes consultation, general practitioners explore further how patients manage their illness burden. We further suggest that diabetes guidelines extend their current focus on clinical and social aspects of diabetes to include questions on patient’s perceived illness burden and SRH.

**Electronic supplementary material:**

The online version of this article (doi:10.1186/s12889-015-1790-6) contains supplementary material, which is available to authorized users.

## Background

General practitioners’ (GPs) and patients’ evaluation of the patient’s health may differ [1-4]. The patient’s own health perception, measured by a single question, known as self-rated general health (SRH), has been shown to predict future morbidity, use of health services, and mortality [5-7]. In patients newly diagnosed with type 2 diabetes (T2DM) seen in general practice, we found an increased 5-year mortality independent of established risk factors among those who rated their health less than excellent [8].

Major decisions about lifestyle changes and treatments are made based on characteristics measured shortly after diabetes diagnosis. It is suggested that the first year after diagnosis of T2DM is both emotionally and practically turbulent for patients [9-11] who have to manage and implement the treatment regimen in their everyday lives [9,11-13].This effort may impact patients’ SRH [14-17]. Research has shown that SRH predicts which patients have a higher risk of diabetic complications, even after accounting for established risk factors like haemoglobin A1c (HbA1c), a marker of glycaemic control [18]. The association between HbA1c and perceived health is not strong [19,20]; indeed, some studies found no association at all [21-23]. The lack of a strong association between HbA1c and perceived health may illustrate that health improvements depend not only on the amelioration of signs and symptoms of hyperglycaemia, but also on other factors. SRH is found to vary with socio-demographic factors [7,24-26], social support [27,28], adaptation and coping with change in objective health [5,7,16,29,30], physical activity level [16], diabetes-related symptoms [20], antidiabetic medication [31], and diabetic complications such as cardiovascular disease (CVD) and neuropathy [32-34].

Few studies have examined change in SRH and its association with health conditions [14-17,35-39], and very few include patients with T2DM [40,41]. In some studies a worsening of health conditions was related to a decline in SRH [14,35-38], but in others this worsening was only related to a weak decline or no change at all [15-17]. This latter finding may illustrate that individuals’ ability to adapt to or cope with objective health change is also important [9,12,16]. Knowledge about factors related to change in SRH could motivate doctors to discuss perceptions of health with newly diagnosed diabetic patients and to be attentive to patients with suboptimal health ratings. Accordingly, there is a need for further research into the relationship between health conditions and changes in SRH.

The aim of this article therefore is: a) to describe the change in SRH from diagnosis until one year later in patients with T2DM seen in general practice, and b) to investigate the association between changes in SRH and coping-related factors, health behaviour, social support, and treatment.

## Methods

This follow-up study includes the intervention group of patients who participated in the Danish randomised trial “Diabetes Care in General Practice” [42], and it covers their first year with a T2DM diagnosis.

### Study population

**Figure 1:**
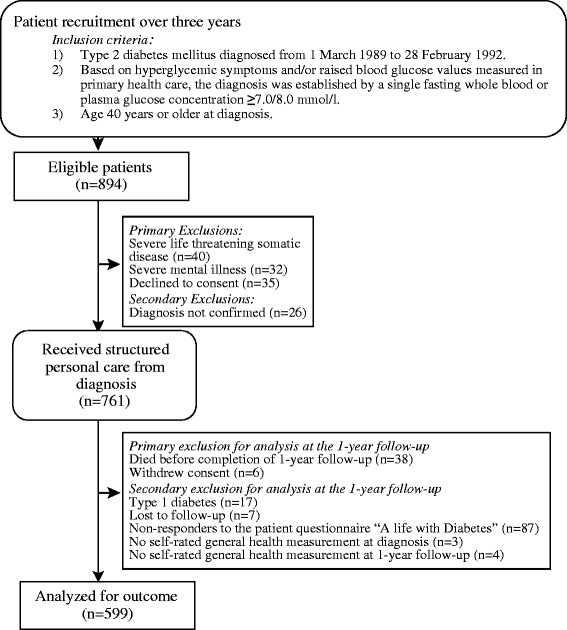
Patient flow through study.

From 1989–1992 the 243 GPs who were part of the intervention group included all newly diagnosed patients with T2DM on their patient list [42] (Figure 1). Of 894 eligible patients, 761 patients remained in the study. Of these, 44 died or withdrew consent before the 1-year follow-up. From the present analysis a further 118 patients were excluded, e.g. due to no response to a central questionnaire. The remaining patients aged 40 to 93 years totalled 599. At diagnosis these 599 patients were older than the 118 patients who were excluded (median age 65.7 vs. 61.7; P = 0.01). No other differences were shown for sex, physical activity, symptoms, or any other factors relevant to SRH.

### Intervention

The GPs in the intervention group were instructed to give structured personal care to the newly diagnosed T2DM patients, which included quarterly consultations and individualized goal-setting, supported by prompting of doctors, printed clinical guidelines, and feedback on individual patients [42].The Copenhagen and Frederiksberg Research Ethics Committee approved the study and all patients gave informed consent.

### Measurements

At diagnosis the GPs recorded height and weight, examined both legs for amputations, presence of patellar reflexes, sense of touch of cotton wool and pin prick, presence of dorsalis pedis or posterior tibial pulse, and recorded history of myocardial infarction and/or stroke causing hospitalization [42]. A fasting blood sample was drawn and a freshly voided morning urine sample was collected. Retinopathy was assessed by practicing ophthalmologists. The centralized methods used for measurement of blood samples and urinary albumin concentration have previously been reported [42]. Fraction of HbA1c was analyzed by ion exchange high-performance liquid chromatography (reference interval: 5.4-7.4%; the interval may cautiously be translated into 4.8-6.7% using a DCCT-aligned method). Only samples measured within 90 days after diabetes diagnosis were accepted.

The GPs, together with the patients, completed a questionnaire regarding the presence of 16 typical symptoms of diabetes (abnormal thirst, frequent urination, unintended weight loss, fatigue, confusion, visual disturbances, cramp in calves (pain or paresthesia in lower extremities), genital itching, balanitis, recurrent urinary tract infections, stomatitis, recurrent skin infections, foot ulcer, gangrene, angina pectoris, intermittent claudication) and one open category. The questionnaire was based on a literature search and interviews with experienced diabetologists. In questionnaires, patients gave information about angina pectoris, intermittent claudication, cancer disease, lifestyle, socio-demographic factors and SRH [42]. SRH was measured with a single validated question [5,43] (see Additional file 1) which was completed a median (interquartile range) of 12 (5–25) days after diabetes diagnosis.

At the 1-year follow-up GPs handed out a patient questionnaire called “A life with Diabetes” (no reminder was issued). The questionnaire was constructed by the study coordinator, Niels Olivarius, and an experienced sociologist, Dorte Gannik. It was based on: a) a literature review of previous qualitative and quantitative research about patients’ views on living with chronic illness; b) theories about living with a chronic illness drawing their main inspiration from the symbolic interactionist theory; c) a review of existing instruments; d) in-depth qualitative interviews with three newly diagnosed patients with T2DM (performed by Dorte Gannik), and e) discussions with health professionals. A group of people familiar with the construction of questionnaires (GPs and sociologists) reviewed the questionnaire several times. Questions were rephrased and/or removed in cases of unclear wording or nonexclusive response categories. After the questionnaire review, 15 T2DM patients tested the questionnaire, and this led to a further revision. Before the questionnaire was sent to the GPs participating in our study, it was shown again to GPs and sociologists familiar with the construction of questionnaires. They had no further comments on the wording and response categories. The questionnaire contained questions on SRH and health behaviour (the patients’ dietary habits and their indication of change in their way of living after diagnosis). In multiple response questions the patients reported whether they received the necessary support and understanding from family and significant others (social support) and how they were coping (two questions exploring whether the patients felt diabetes was a mental/practical and/or an illness burden in daily life, and their emotional attitudes towards diabetes). The responses concerning social support and coping were summarised into new categories (see Additional file 1). The GPs reported on the patients’ antidiabetic treatment and the number of diabetes-related consultations within the last year.

### Definitions

A symptom score was constructed by adding together positive answers to the symptom questions. The open category was coded as “other symptoms”. Body mass index (BMI) was calculated as (weight in kg)/(height in m)^2^. Peripheral neuropathy (neuropathy) was defined as lack of a sense of pin prick and/or touch of cotton wool on at least one foot and/or absent patellar reflex on at least one knee. Urinary albumin concentration was used to define microalbuminuria (≥15 - <200 mg/ml) and proteinuria (≥200 mg/l). CVD was defined as history of myocardial infarction and/or history of stroke and/or angina pectoris and/or intermittent claudication and/or absent arterial pulses on both feet and/or amputation of the lower extremities. Diabetic retinopathy was defined as presence of at least one microaneurysm.

### Statistical analysis

First, the relation between SRH (we combined the two lowest categories, poor and very poor, into one category, “poor”, due to few respondents reporting very poor) and socio-demographic status, diagnostic HbA1c, and other factors indicating the illness severity at diagnosis (Table 1) were analysed bivariately to establish the baseline distribution of SRH. One year later we made a similar comparison between SRH and the patients’ indication of coping, health behaviour, social support, and treatment since these variables are suggested to vary with SRH (Table 2). Illness severity at diagnosis may possibly have influenced the variation of coping, health behaviour, social support, and treatment. Those patients with diabetic complications would hence be more likely to have another health behaviour than those without complications, and therefore our analysis took account of the distribution of these factors (see below). Factors relevant to glycaemic control, like diabetic complications and cardiovascular status, were not measured one year after diagnosis as there would be little meaning in obtaining such information after only one year. Moreover, most patients with T2DM are still in the early stages of disease progression “honeymoon” period one year after diagnosis, and the blood glucose level is typically very low [44].

**Table 1:** **Distribution of self-rated general health by patient characteristics at diabetes diagnosis in patients with type 2 diabetes**

	**Self-rated health**				**P**
**Characteristics**	**Very good**	**Good**	**Fair**	**Poor**	
N (%)	71 (11.6)	210 (35.1)	267 (44.6)	51 (8.5)	
Sex (%)					0.18
Women	28 (39.4)	93 (44.3)	141 (48.8)	27 (52.9)	
Men	43 (60.6)	117 (55.7)	126 (47.2)	24 (47.1)	
Age (Years)	65.5	65.9	65.9	63.5	0.59
	(56.0-73.7)	(54.1-73.2)	(57.4-74.4)	(57.1-73.1)	
Cohabiting (%)					0.64
Yes	48 (68.6)	151 (71.9)	185 (69.3)	32 (62.8)	
Smoking habits (%)					0.72
Never	20 (28.2)	70 (33.7)	82 (30.7)	18 (35.3)	
Former	27 (38.0)	73 (35.1)	83 (31.1)	17 (33.3)	
Current	24 (33.8)	65 (31.3)	102 (38.2)	16 (31.4)	
Level of physical activity (%)					<0.0001
Much	8 (11.4)	24 (11.3)	10 (3.8)	1 (2.0)	
Moderate	51 (72.9)	145 (69.1)	170 (63.9)	27 (52.9)	
Low	11 (15.7)	41 (19.5)	86 (32.3)	23 (45.1)	
Number of diabetes-related symptoms^a^	3	3	4	5	<0.0001
	(1–4)	(2–5)	(2–5)	(3–7)	
Haemoglobin A1c^b^ (fract., %)	9.6	9.9	9.6	9.1	0.53
	(8.0-11.3)	(8.5-11.5)	(8.1-11.5)	(8.2-11.2)	
Body mass index (kg/m^2^)	28.9	29.4	29.4	29.2	0.49
	(26.0-32.2)	(26.2-32.6)	(26.3-32.9)	(25.8-33.6)	
Cancer (%)					0.73
Yes	4 (5.7)	8 (3.8)	20 (7.5)	5 (9.8)	
Complications (%)					
Retinopathy					0.72
Yes	3 (4.4)	11 (5.7)	9 (3.7)	3 (6.4)	
Neuropathy					0.64
Yes	12 (17.1)	39 (18.8)	48 (18.2)	13 (29.5)	
CVD					<0.0001
Yes	9 (12.7)	46 (21.9)	90 (33.7)	25 (49.0)	
Urinary albumin					0.40
Normal	37 (55.2)	117 (60.6)	132 (55.7)	23 (52.3)	
Microalbuminuria	27 (40.3)	72 (37.3)	89 (37.6)	19 (43.2)	
Proteinuria	3 (4.5)	4 (2.1)	16 (6.8)	2 (4.6)	

Values are numbers (%) or medians (interquartile range). P-values are from Kruskal Wallis tests and χ^2^-tests. CVD, cardiovascular disease.

^a^Symptom score was constructed by adding number of reported diabetes-related symptoms at diagnosis. The response categories consisted of 16 predefined symptoms and an open response category: abnormal thirst, frequent urination, unintended weight loss, fatigue, confusion, visual disturbances, cramp in calves (pain or paresthesia in lower extremities), genital itching, balanitis, recurrent urinary tract infections, stomatitis, recurrent skin infections, foot ulcer, gangrene, angina pectoris, intermittent claudication, and other symptoms.

^b^Reference interval: 5.4 – 7.4%.

**Table 2:** **Distribution of self-rated general health by patient characteristics one year after diagnosis of type 2 diabetes**

**Characteristics**	**Self-rated health**				**P**
	**Very good**	**Good**	**Fair**	**Poor**	
N (%)	131 (21.9)	263 (43.9)	186 (31.1)	19 (3.2)	
Sex (%)					0.52
Women	59 (45.0)	123 (46.8)	98 (52.7)	9 (47.4)	
Men	72 (55.0)	140 (53.2)	88 (47.3)	10 (52.6)	
*Health behaviour:*					
Has changed way of living after diagnosis (%)					0.22
Yes	99 (76.2)	211(82.4)	153 (84.5)	16 (88.9)	
Food habits (%)					0.055
Diabetes diet^a^	57 (44.2)	124 (49.0)	82 (46.3)	8 (42.1)	
Full diet without sugar	50 (38.8)	110 (43.5)	79 (44.6)	19 (57.9)	
Diet as non-diabetics	22 (17.1)	19 (7.5)	16 (9.0)	0	
General practice visits per year	3.7	3.5	3.7	3.6	0.51
	(2.6-4.8)	(2.6-4.7)	(2.8-5.0)	(2.7-4.3)	
*Treatment:* Diet as only treatment to reduce blood glucose (%)	93 (71.0)	143 (56.8)	91 (51.3)	6 (33.3)	0.0005
*Social support* (%)					0.0002
Full support	92 (73.0)	165 (65.5)	93 (52.8)	9 (50.0)	
Handles it myself	26 (20.6)	71 (28.2)	54 (30.7)	4 (22.2)	
Feel alone, misunderstood	8 (6.4)	16 (6.4)	29 (16.5)	5 (27.8)	
*Coping:*					
Illness burden (%)					<0.0001
No burden	85 (68.0)	139 (59.1)	64 (36.0)	1 (5.3)	
Minor burden	30 (24.0)	68 (26.5)	56 (31.5)	5 (26.3)	
Some burden	9 (7.2)	45 (17.5)	40 (22.5)	5 (26.3)	
Major burden	1 (0.8)	5 (2.0)	18 (10.1)	8 (42.1)	
Attitudes towards diabetes (%)					<0.0001
The illness is unproblematic^b^	49 (38.0)	113 (43.6)	81 (44.3)	4 (23.5)	
Work/have worked with the illness^c^	78 (60.5)	134 (51.7)	71 (38.8)	5 (29.4)	
It is a strain	2 (1.6)	12 (4.6)	31 (16.9)	8 (47.1)	

Values are numbers (%) or medians (interquartile range). P-values are from Kruskal Wallis tests and χ^2^-tests.

^a^Diet with certain amounts of selected foodstuffs.

^b^Life is not altered/the illness is unproblematic.

^c^Work/have worked with the illness in order to cope or adapt.

Variables indicating the patients’ coping strategies and social support are regarded as latent variables. To ensure that a co-variation between SRH and these variables does not reflect a common underlying latent variable, we performed a graphical Rasch analysis [45] conditional on the exogenous variables age, diagnostic HbA1c, smoking habits, and physical activity.

With a multivariate analysis we estimated how the covariates influenced change in SRH from diagnosis until one year later. Illness severity at diagnosis may influence the variation of the covariates: patients with diabetic complications may have health behaviours which are different from those without complications, and an analysis accounted for the distribution of these factors.

The expected level of the two SRH measurements was modelled using a cumulative probit model for ordinal data. One interpretation of this model is that the ordinal measure of SRH is the realization of a continuous, latent SRH, assumed to be normally distributed, with a mean that may be allowed to depend on the covariates, and a variance of one. The larger this latent SRH, the better the SRH. The expected differences in the person’s level of SRH at diagnosis were estimated using baseline information. To avoid a scenario where an effect of a covariate on the level of SRH at diagnosis was misinterpreted as an effect on the estimated change between the two time points, the covariates were also used for modelling the mean SRH at diagnosis. The expected change in SRH, defined as latent SRH one year after diagnosis minus latent SRH at diagnosis, was allowed to depend on the patients’ indication of the impact of diabetes on daily life and their evaluation of social support, indication of change in their way of living, and other variables shown to be significant for the physical condition of diabetes and life in general. (These are age, sex, cohabiting status, smoking habits, physical activity, number of diabetes-related symptoms, HbA1c, BMI, cancer, peripheral neuropathy, diabetic retinopathy, CVD, urinary albumin, change in way of living after diagnosis, food habits, clinic visits per year, antidiabetic treatment, social support, illness burden, and attitudes towards diabetes). The test of whether a variable relates to change in SRH corresponds to a test of interaction with time in the cumulative probit model.

A patient’s SRH at two different time points may be correlated. The analysis therefore took account of a possible correlation of measurements within one person to avoid possible incorrect conventional confidence intervals for the different parameters by using generalized estimating equation methods (PROC GENMOD, SAS version 8.2). The hypotheses were tested using a generalized Wald test. Hypotheses regarding the effect of ordinal, categorical variables on change in latent SRH or at the level of latent SRH at diagnosis were tested as a trend test using a model where the ordinal variable was included as a continuous variable. In the multivariate analyses we performed backwards elimination of variables based on p ≤ 0.25. The nominal statistical significance level was <0.05.

## Results

At diagnosis, 11.6% of patients reported very good, 35.1% good, 44.6% fair, and 8.5% poor SRH. Only a few variables were related to SRH, but the number of symptoms increased with decreasing SRH (Table 1). Likewise, low physical activity level and CVD were associated with poor SRH.

One year after diagnosis, in a univariate analysis, patients tended to rate their health better when diet was the only treatment instituted to lower blood glucose (Table 2). Patients’ indications of social support were closely related to SRH: the poorer the health ratings, the fewer patients indicated full support. Both perceived illness burden and attitudes towards diabetes were related to SRH, e.g. major illness burden and the attitude “the illness is a strain” reflected poorer health ratings. The graphical Rasch analysis revealed, however, that SRH, social support, attitudes towards diabetes, and perceived illness burden did not reflect an underlying latent variable: the responses on each variable did not have an identical pattern. Small local dependencies were found between SRH and the three variables, but none of the variables were regarded as identical with SRH (data not shown).

The multivariate analysis of change (without covariates) showed that patients on average experienced an SRH increase of 0.46 (95% CI: 0.37; 0.55(data not shown)) during the first year after diabetes diagnosis. After inclusion of covariates, only illness burden was associated with a change in SRH after backward elimination of non-significant associations (Table 3, complete data existed for 583 patients). The estimates in Table 3 should be interpreted as the expected difference in the change of latent SRH between a patient from the relevant group and a patient from the reference group (where the estimate equals zero) with the two patients having similar values for all other included covariates. Patients with a major illness burden on average experienced a 0.56 smaller increase in latent SRH than the increase in SRH among otherwise similar patients who reported no illness burden at all (= 0).

**Table 3:** **Self-rated general health change vs. illness severity and patient experience one year later. Multivariate analysis**

	**Difference in change in SRH** ^**a**^	**95% CI**	**P**
**Illness severity at diabetes diagnosis:**			
CVD^b^			
Yes	0.23	−0.05; 0.51	0.11
No	0	-	
Peripheral neuropathy			
Yes	−0.30	−0.64; 0.04	0.09
No	0	-	
Urinary albumin mg/ml			
Proteinuria: ≥ 200	−0.12	−0.65; 0.41	0.06 ^c^
Microalbuminuria: ≥15- < 200	−0.30	−0.55; −0.05	
Normal: < 15	0	-	
Diabetes-related symptoms			
K + 1	0.04	−0.01; 0.10	0.14
K	0	-	
**Patient experience at one year follow-up:**			
*Health behaviour:*			
Has not changed way of living after diagnosis	0.21	−0.11; 0.53	0.20
Has not changed way of living after diagnosis	0		
General practice visits per year			
K + 1	0.05	−0.03; 0.12	0.20
K	0	-	
*Social support:*			
Feel alone, misunderstood	−0.33	−0.76; 0.09	0.10 ^c^
Handles it myself	−0.13	−0.40; 0.14	
Full support	0	-	
*Coping:* Illness burden			
Major burden	−0.56	−1.19; 0.06	0.03 ^c^
Some burden	−0.26	−0.60; 0.07	
Minor burden	−0.07	−0.35; 0.21	
No burden	0	-	

^a^Self-rated general health.

^b^CVD, cardiovascular disease.

^c^Trend test.

## Discussion

This follow-up study demonstrates an average improvement in patients’ SRH during their first year following a diagnosis of T2DM. At diagnosis poor SRH was associated with low physical activity, presence of CVD, and many diabetes-related symptoms. One year after diagnosis better SRH-ratings were associated with full support by family and friends, no illness burden, and finding the illness to be unproblematic. In multivariate analyses only the patients’ perceived illness burden was associated with the change in SRH during the first year: those who indicated that the illness was a burden had an estimated smaller increase of SRH from time of diagnosis until one year follow-up, compared with otherwise similar patients who stated no illness burden at all. Socio-demographic factors or illness severity had no impact on change in SRH.

### Study strengths and limitations

The major study strength is the population-based sample of patients with T2DM treated in general practice and examined at two well-defined points of time in the natural history of diabetes. We modelled change in SRH by use of the absolute difference in latent SRH. It is not obvious how a difference in SRH should be modelled. Some authors [15,39] have analyzed the absolute difference in SRH, while others have defined change as change at two levels on e.g. a five-point SRH scale, with exceptions from the middle category [46,47]. This method disregards that a difference in SRH of one may not have the same impact on all SRH categories. Instead we chose to model change in SRH by use of the absolute difference in latent SRH, since SRH can be regarded as a latent variable which may vary in a continuous manner. The latter method makes it very simple to allow it to depend on covariates. Another study strength was the use of graphical Rasch analysis which revealed that the coping factor in our study, illness burden, and SRH could not be regarded as reflecting an identical underlying latent variable. It is therefore unlikely that illness burden is a proxy measure of SRH.

One limitation of this study was the use of self-reported questionnaire data. Patients may have overestimated their actual behaviour to provide a socially desirable response [48,49]. Another limitation is that we do not know whether change in SRH is due to the patients adapting to their illness, or whether the patients experienced an actual change in health status. We only included information on illness severity at diagnosis. This is not likely to have influenced our results since it would give little meaning to obtain such information again after only one year. Furthermore, we included only the surviving patients and those patients who responded to the “A life with Diabetes” questionnaire one year after diagnosis. It is likely that baseline SRH would have been lower, and that overall changes in SRH from diagnosis until one year later would be lower than 0.46 if we had included non-survivors and non-respondents in the analysis, however non-responders had similar values on factors relevant to SRH and it is likely that inclusion of their information would not have had a significant impact on the overall changes in SRH.

The study is limited by having only two measurements of SRH and any interpretations of causality should be carefully approached.

### Our results in relation to other studies

Our study confirms results from previous studies showing an association between poor SRH rating and: 1) a low level of physical activity [16,24,39,50], and 2) the presence of many diabetes-related symptoms. [19,20,51] It is suggested that the relation between HbA1c and SRH is mediated by symptom perception, which tends to be rather individual [52-54], and this may contribute to explaining the lack of association between HbA1c and SRH. CVD was related to poor SRH, but contrary to earlier studies [18,32,33] other diabetic complications did not impact SRH, possibly due to the short duration of diabetes in our study.

Our cross-sectional analysis one year after diabetes diagnosis tallies with the results obtained from the American Centers for Disease Control and Prevention [41] and Jacobson et al. [31] who found poor SRH-ratings among patients receiving antidiabetic medication. However, in our study this medication was not related to change in SRH, possibly because the association was expressed through complications which are more prevalent in patients receiving antidiabetic medication. The importance of social support on perceived health among patients with T2DM has also been found in other studies [9,55,56], and social support may possibly buffer stressful life events so that perceived health may not be affected. Kelleher [57] studied compliance in adults with diabetes and found, as we did, that negative illness attitudes were related to poor perceived health, and that people worrying about their illness felt unhealthy, even if they followed the treatment.

Only the patients’ perceived illness burden was associated with SRH change in our study. The lack of association between change in SRH and any indicator of objective health status, health practices, or utilization of health services confirms the results from an American study. This study included middle-aged people where health indicators were related to actual SRH but not to change in SRH over a 1-year period [17]. For example, in our study a low level of e.g. physical activity at diagnosis was related to a relatively poor SRH-rating compared to patients with a higher activity level, but not to change in SRH. In contrast, a 3-year study by Rodin and McAvay among elderly people (>62 years) with nine measurements of SRH found that a relative decline in SRH between two time-points was related to worsening of pre-existing conditions or new illnesses [14]. Leinonen et al. studied change with advanced statistical techniques in a 5-year [15] and a 10-year [16] follow-up including two and three SRH measurements of 75-year-old Finnish people (at baseline). They concluded in their 5-year follow-up that no big SRH changes were found compared with baseline, even though objective health declines were found. At the 10-year follow-up a systematic association between SRH and relevant covariates was only present among people who either had poor or good SRH between the 5-year and 10-year periods. The latter may show that “objective” health changes may not be closely related to a person’s SRH ratings, which we also found in our study. As Leinonen et al. and Jylhä state, this may be due to better adaptation or coping strategies for physical, mental, or social decline when growing older [7,16].Elderly people may in general expect to have health problems, and elderly people diagnosed with new chronic conditions have shown no decrease in their SRH compared to more healthy people diagnosed with new chronic conditions [7].

In some studies medical treatment and lifestyle changes to accommodate to a disease have been shown to have less impact on people’s perceived health than would be expected from an evaluation of other health indicators [15,58,59]. This possibly indicates, as Leinonen et al. found in their 10-year follow-up [16], that people may view illness and treatment as something to be expected, as a part of growing old, or as a result of their lifestyle or family history. Studying how people learn to live with a chronic illness, Strauss et al. [9] found that people who were facing problems in their lives in general, also more easily accepted the diagnosis of a disease and tried to incorporate changes in their daily lives. Their strategy was to accommodate the changes and they neither perceived much treatment burden nor found that their internal biography was as much disrupted by the illness as people with a lower coping capacity. Some people have a greater capacity to cope or have the resources to change the situation in their favour, if necessary, while other people have not [60]. The latter group shows the relevance of paying attention to how individual patients cope with an illness in their particular situation, and to their personal health resources. Studies in general practice relating to the dialogue about patients’ health resources have developed key questions about patients’ strengths, and their answers include valuable information that can be used for further treatment initiatives [61].

Jylhä also recommends that GPs use SRH as a kind of screening tool for patients’ health status which could be interpreted together with other measurements [7]. Good SRH is not a guarantee of physician-evaluated good health, however, when patients rate their health as poor, this is information that should lead to further investigation of what lies behind the patient’s evaluation [7]. Furthermore, our study suggests that GPs have to be aware that improved glycaemic control, which is a primary focus in guidelines for diabetes treatment in order to decrease the risk of diabetic complications, is not necessarily followed by an increase in SRH [20,62]. Treatment burden and self care demands may impact the patients’ evaluation of their own health. It is important that GPs negotiate treatment goals with the patients in order to balance the impact of the treatment regime with the patients’ lifestyles and their coping styles.

## Conclusions

During the first year after diagnosis of T2DM SRH improves for many patients, and their perceived illness burden seems to be associated with the change in SRH. The causal direction is unknown, but the increase in SRH was lower among patients with a greater illness burden. However, change in SRH was not associated with indicators of illness severity or factors reflecting socio-demographic circumstances.

The diabetes consultation gives the GP and other health professionals an opportunity to gain knowledge about how the individual patient tolerates and manages the illness burden and it can bring to light those personal health resources that may potentially minimize this burden. Current guidelines recommend that clinical and social aspects of diabetes are considered by the GP in an effort to optimize treatment. Our results indicate that it may also be relevant to extend these guidelines with simple key questions about the patient’s SRH and personal perception of the illness burden.

## Additional file

Additional file 1:
**Item wording and coding categories for selected questions.**

## Abbreviations

BMI: Body mass index
CVD: Cardiovascular disease
GP: General practitioner
HbA1c: Haemoglobin A1c
Neuropathy: Peripheral neuropathy
SRH: Self-rated general health
T2DM: type 2diabetes
