# Supplementary material for: Change in self-rated general health is associated with perceived illness burden: a 1-year follow up of patients newly diagnosed with type 2 diabetes
Source: BMC Public Health. 2015 Apr 30;15:439. doi: 10.1186/s12889-015-1790-6 (PMC4431173; doi:10.1186/s12889-015-1790-6)
Supplement: Additional file 1: — Item wording and coding categories for selected questions. [file 12889_2015_1790_MOESM1_ESM.docx]

| **Additional file 1**  **Item wording and coding categories for selected questions** | | | |
| --- | --- | --- | --- |
| Variable name | Item wording | Response categories | Coding categories |
| Self-rated health^*^ | In general, how would you rate your health at present? | 1. Very good 2. Good 3. Fair 4. Poor 5. Very poor | Very good = 1  Good = 2  Fair = 3  Poor = 4 and 5^\|\|^ |
| Leisure time physical activity^†^ | If we look back on the past year, what would you say best describes your leisure time activities? | 1. Heavy training and competitive sports regularly and several times a week 2. Exercise or heavy gardening at least 4 hours a week 3. Walk, bike or other easy exercise at least 4 hours a week (include Sunday excursions, light gardening and biking/walking to work) 4. Read, watch TV or other sedentary occupation | High = 1 and 2  Medium = 2  Low = 3 |
| Dietary habits^‡^ | If you have to answer what you really eat, and not what you were told to eat, would you say you: | 1. Eat almost the same as those not having diabetes 2. Eat what I like to, but not sugar and sweets 3. Eat a certain amount of bread, potatoes, milk, and fruit (by eye) 4. Scale/weigh a certain amount of bread, potatoes, milk, and fruit to one or more meals/week | Diet as non-diabetics = 1  Full diet without sugar = 2  Diabetes diet = 3 and 4 |
| Social support^‡§^ | All in all do you feel you get the necessary support and understanding from others (family, friends, work etc.) to handle the illness in daily life? | 1. I feel fully supported 2. Sometimes, I feel no understanding 3. I often feel alone with the problem 4. I can handle it by myself | Full support = (1 alone; 1 + 4 together; 1 + 2 + 4; 1 + 2)  Handle it by one self =  (4; 2 + 4; 2 + 3 + 4; 3 + 4)  Feel alone, misunderstood = (2; 3; 2 + 3) |
| Attitudes towards diabetes^‡§^ | What are your own attitudes towards the illness? | 1. It has not changed much in my life 2. I have learned to live with it 3. It is a challenge that I try to handle 4. It is a strain | Life has not changed = (1; 1 + 2)  Adapted to, work /have worked with the illness = (2; 3; 1 + 3; 1 + 2 + 3; 2 + 3)  It is a strain = (4; 1 + 2 + 3 + 4; 1 + 2 + 4; 1 + 3 + 4; 1 + 4; 3 + 4; 2 + 4) |
| Illness burden ^‡§^ | Do you find it difficult to have diabetes? | 1. Yes, I feel it is a strain every day 2. Yes, I often think about it 3. No, I only think about it once a while 4. No, all in all I feel like I do not have diabetes | No = (4; 3 + 4)  Minor = (3)  Some = (2; 2 + 3; 2 + 4; 2 + 3 + 4)  Major = (1; 1 + 2; 1 + 3) |

^*^ Measured at diabetes diagnosis and one year later.

^†^ Measured at diabetes diagnosis.

^‡^ Measured one year after diabetes diagnosis.

^§^A multiple response question.

^||^Due to the very small number of patients with very poor self-rated health, the two lowest categories, poor and very poor, have been united into one category, “poor”.
